# Effectiveness of a Multimodal Therapy for Patients with Chronic Low Back Pain Regarding Pre-Admission Healthcare Utilization

**DOI:** 10.1371/journal.pone.0143139

**Published:** 2015-11-23

**Authors:** Constanze Borys, Johannes Lutz, Bernhard Strauss, Uwe Altmann

**Affiliations:** 1 University Hospital Jena, Institute of Psychosocial Medicine and Psychotherapy, Friedrich -Schiller-University Jena, Jena, Germany; 2 Interdisciplinary Pain Center, Central Hospital Bad Berka, Bad Berka, Germany; Central South University, CHINA

## Abstract

**Objective:**

The aim of the study was to examine the effectiveness of an intensive inpatient three-week multimodal therapy. We focused especially on the impact on the multimodal therapy outcome of the pre-admission number of treatment types patients had received and of medical specialist groups patients had consulted.

**Methods:**

155 patients with chronic low back pain and indication for multimodal therapy were evaluated with respect to pain intensity, depression, anxiety, well-being, and pre-admission health care utilization. In our controlled clinical trial we compared N = 66 patients on the waiting list with N = 89 patients who received immediate treatment. The waiting list patients likewise attended multimodal therapy after the waiting period. Longitudinal post-treatment data for both were collected at three- and twelve-month follow-ups. The impact of pre-admission health care utilization on multimodal therapy outcome (post) was analysed by structural equation model.

**Results:**

Compared to the control group, multimodal therapy patients’ pain intensity and psychological variables were significantly reduced. Longitudinal effects with respect to pre-measures were significant at three-month follow-up for pain intensity (ES = -0.48), well-being (ES = 0.78), anxiety (ES = -0.33), and depression (ES = -0.30). Effect sizes at twelve-month follow-up were small for anxiety (ES = -0.22), and moderate for general well-being (ES = 0.61). Structural equation model revealed that a higher number of pre-admission treatment types was associated with poorer post-treatment outcomes in pain intensity, well-being, and depression.

**Conclusion:**

Multimodal therapy proved to be effective with regard to improvements in pain intensity, depression, anxiety, and well-being. The association between treatment effect and number of pre-admission pain treatment types suggests that patients would benefit more from attending multimodal therapy in an earlier stage of health care.

## Introduction

Access to outpatient medical services is almost barrier-free in the German health system, that is, without any “gate-keeping-system.” This includes consultations with most medical specialists [1]. In orthopedic practice, low back pain (ICD-10 diagnosis M54) was the most common reason for a consultation in 2013, with 42.9% of cases. In general practice, low back pain is in third place when considering all diseases (14.9%) [2]. Most guidelines recommend the management of non-complicated low back pain in primary care as sufficient therapy [3]. Prolonged, disabling pain should be diagnosed and treated with multimodal therapy [3, 4]. There is empirical evidence for the effects of intensive multimodal therapy (≥100h) using a “functional restoration” approach [5]. Usually, multimodal therapy in Germany is an inpatient therapy and requires referral and an interdisciplinary diagnostic assessment before treatment starts.

The treatment algorithm for managing low back pain first recommends:

- consultation in a multimodal setting for an interdisciplinary assessment after 8–12 weeks of pain,

and/or

- 4–6 weeks out of work due to low back pain,

and/or

- significant indication of a risk of chronicity [6, 7].

In reality, patients in multimodal therapy report suffering from low back pain for many years and reveal a long history of consultations and various treatments. Most of these patients already report seriously reduced physical and emotional functioning due to low back pain [8, 9].

Existing studies revealed several predictors of reduced pain intensity in patients with chronic low back pain following multimodal Therapy (MMT) [10, 11]. Predictors were average pain intensity, improved functional capacity due to treatment, affective distress, and shorter absences from work. Furthermore, pre-admission healthcare utilization predicts the success of MMT. Hildebrandt et al. [10], for example, reported an association between medical consultations before MMT and reduced pain intensity one year after MMT. Likewise, in the study of Gross & Battie [12], the number of pre-admission healthcare visits was the most robust predictor of delayed recovery and recurrence of low back pain.

The present study aims to evaluate an intensive, three-week inpatient MMT for patients with chronic low back pain, focusing on the impact that the amount of healthcare utilization has on the outcome of MMT. The aims and hypotheses are:

1. Evaluating the effectiveness of MMT: Compared to waiting group, patients receiving MMT were expected to improve significantly from pre- to post-treatment.
2. Prospective evaluation at three- and twelve-month follow-ups: We hypothesized that patients would improve significantly in pain-related and psychological variables following MMT. We expected those improvements to decrease by three- and twelve-month follow-up intervals.
3. Impact of healthcare utilization on MMT outcome: We hypothesized that the amount of pre-admission healthcare utilization would predict responses to MMT. We expected that more utilization leads to less improvement.

## Materials and Methods

Prior to the investigation, all participants gave written informed consent. The study was approved by the local ethics committee (Kassenaerztliche Vereinigung Weimar, 22825/2010/117).

### Study design

Assessments were conducted at the beginning of the waiting time (t_0_), the beginning of MMT (t_1_), the end of MMT (t_2_), and three (t_3_) and twelve months (t_4_) to follow up. Analyses for the first hypothesis were based on a pre-post-two-group design. The control group (waiting group patients, N = 66) was compared with immediately-treated patients (intervention group, N = 89). The outcome measures were assessed at the end of the waiting period (t_1_) for the waiting group and at the end of MMT (t_2_) for the group receiving treatment immediately. As the pre-test we used t_0_ (control group) resp. t_1_ (intervention group). The analysis for the second hypothesis was based on a one-group longitudinal design. Accordingly, all time points were considered and all patients (waiting group and immediately-treated patients, N = 155) were included in the statistical analysis.

To analyze the impact of healthcare utilization on MMT outcome (third hypothesis) the intervals t_1_ (pre-MMT-treatment) and t_2_ (post-MMT-treatment) of all patients (N = 155) were considered. Pre-admission healthcare utilization, pre-treatment pain intensity, depression, anxiety, and well-being served as predictors for post-treatment pain intensity, depression, anxiety, and well-being.

### Subjects

#### Inclusion and exclusion criteria

Participants were recruited in a regular, naturalistic, German pain management setting. Criteria for participation were: (1) chronic (duration >3 months) low back pain as predominant pain condition, (2) indication for attending MMT, (3) sufficient understanding of the German language, and (4) aged between 18 and 70 years. Indication criteria for MMT were based on the German guidelines for managing non-specific low back pain. During a three-day inpatient pre-treatment multi-disciplinary pain assessment the indication was verified by an interdisciplinary team (pain physicians, psychotherapists, physiotherapists, and pain nurses).

#### Sample

**Fig 1:**
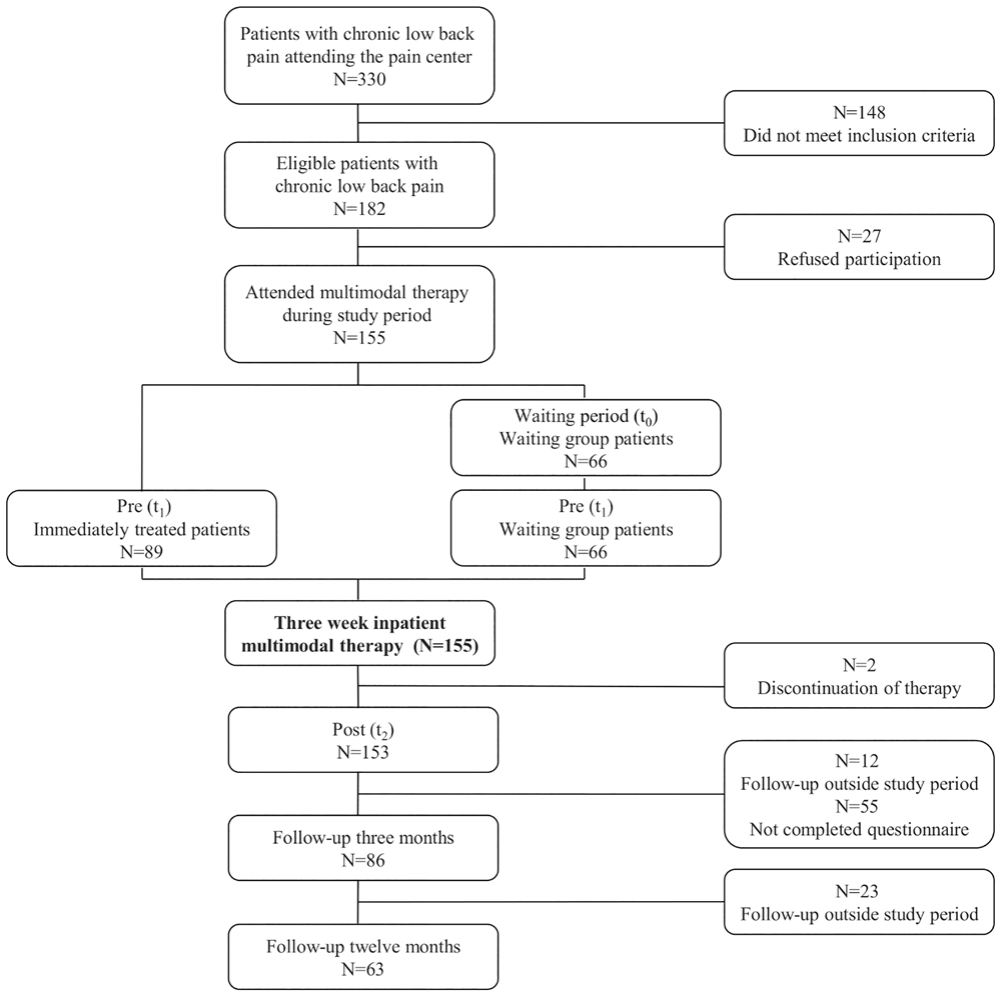
Flowchart with number of participants and dropouts for baseline, pre-treatment, post-treatment and follow-up at three and twelve months.

N = 330 patients with chronic low back pain attended the pain center ***w***ithin the study period (2008 to 2011); 182 patients proved to be eligible for MMT. Twenty-seven patients refused to sign the informed consent and were excluded. Finally, 155 patients were included in the study. Of these, sixty-six patients had a waiting period (waiting group) of at least three months prior to MMT. Patients waited for different reasons (multimodal therapy currently not available, further assessment necessary etc.). Treatment as usual was carried out during the waiting period. Eighty-nine patients were treated immediately with MMT. Fig 1 illustrates the selection process and resulting sample sizes for each assessment interval. Descriptive sample statistics are presented in the results section.

### Procedure

#### Interdisciplinary pain center

The Central Hospital in Bad Berka, Germany provides inpatient pre-treatment multi-disciplinary pain assessment (three days) and, if indicated, an intensive, three-week inpatient MMT. The main aim of the MMT was to improve functional capacity, reduce pain intensity, improve emotional functioning and well-being, and increase the patients’ knowledge of bio-psycho-social pain mechanisms and pain-related coping-strategies.

An interdisciplinary therapeutic team (physicians, clinical psychologists, physiotherapists, and pain nurses) performed an individually-adjusted treatment within a standard schedule (eight hours a day, six days a week). Regular team meetings for the evaluation of individual treatment progress were carried out. Medical treatment consisted of modification of analgesic medication and also frequently withdrawal of medication in case of lack of effectiveness or an increasing burden of side effects. Behavioral management included education (pain and psychological factors), biofeedback and cognitive-behavioral therapy (group therapy once a day, individual therapy once a week) offered by psychologists. Furthermore, patients received physiotherapy (physical exercise and “work hardening”) and active training sessions (e.g., Nordic walking) offered by trained physiotherapists.

All patients of the MMT group underwent pre-treatment multidisciplinary pain assessment. This included physical examination, functional testing, and further investigations to exclude red flags (e.g. MRI-scan, second opinion of orthopedic-neurological and neurosurgical physician). Moreover psychological-, psychosomatic- or psychiatric diagnostic factors have been evaluated. The assessment team consisted of physicians (specialized in pain management), psychologists/psychotherapists, pain nurses, and physiotherapists. Based on the medical and psychological findings, an individualized treatment was recommended by the assessment team.

### Materials

The primary outcome was pain intensity (assessed using NRS, see below). Secondary outcomes were depression, anxiety and well-being (assessed using ADS, HADS, and MFHW, respectively, see below).

*German Pain Questionnaire of the German Section of the IASP* (DSF: [13]). The DSF was used for assessing socio-demographic and pain-related characteristics.

*Numeric Rating Scale (NRS)*. Pain intensity was assessed by the NRS 0–10 (0 = “no pain at all” to 10 = “strongest imaginable pain”).

*Pain duration*. The duration of pain was assessed as the number of months since the first appearance of back pain.

*Number of consulted medical specialist groups*. Participants were asked to indicate the use of specialists for their back pain symptoms from a list of 11 medical specialist groups (e.g., general physician, neurologist, orthopedist, psychiatrist, and pain therapist) [13].

*Number of types of treatment used*. Additionally, participants had to indicate the use of different treatment types out of a list of 13 different treatments (e.g., massage, medication, and body exercises) [13].

The assessment of psychological characteristics was carried out using standardized, established questionnaires with adequate to good psychometric characteristics (see below).

*General Depression Scale (ADS*: [14]*)*. The ADS is a self-report scale for assessing depressive symptoms in their emotional, motivational, cognitive, somatic, and interpersonal manifestation. The short form used for this study contains 15 items (4-point Likert-scale). Sum scores of ≤ 22 are considered normal [14].

*Hospital Anxiety and Depression Scale* (HADS: [15], German version:[16]). The HADS was developed to determine the levels of anxiety and depression in individuals with physical health problems. Only the anxiety scale was used for the present study to avoid highly-correlated covariates. As a depression scale we used the ADS instead (see above). The HADS anxiety scale contains seven items (4-point Likert-scale). Sum scores of ≤ 7 are considered normal [16].

*Marburg Questionnaire of Habitual Well-Being* (MFHW: [17]). The MFHW is a one-dimensional questionnaire to assess positive characteristics of well-being (e.g., “I can enjoy my life”). It comprises seven items (5-point Likert-scale). The maximum sum score of 35 indicates high levels of well-being. Sum scores ≤ 10 within pain patients are rated as abnormal [17].

Socio-demographic characteristics, pain duration, and pre-admission healthcare utilization were assessed once at the first consultation at the pain center. Pain intensity and all psychological variables were surveyed at all five assessment intervals.

#### Dropout

Overall, two of the N = 155 patients terminated their treatment prematurely. Ninety patients did not complete the documentation until the twelve-month follow-up, but completed the treatment program. A comparison of all ninety (58.1%) dropouts vs. completers revealed no systematic differences in socio-demographic, pain-related, psychological basic variables, and healthcare utilization.

### Missing data

In our study, we had a moderate proportion of missing data. Socio-demographic variables were complete. Scores of outcome variables (NRS, MFHW, HADS, and ADS) were completed at t_1_ (when therapy began). Healthcare utilization variables had few missing values: eleven of the 155 patients revealed no information about pre-admission consultations with medical specialists. Five patients had received different treatment types before MMT without specifying the number. Long-term treatment with opioids was unclear in one case. We tested the “missing complete at random (MCAR)” hypothesis using socio-demographic variables, the four outcome variables at the assessment time points t_0_, t_1_, t_2_, t_3_, and t_4_, and healthcare utilization variables. The null hypothesis could not be rejected (Little’s MCAR-test Chi^2^ = 637.5, df = 644, p = .564). This suggests “MCAR.” We applied the full-information-maximum-likelihood estimator (FIML) which produces unbiased parameter estimates and standard errors under “MCAR” and “missing at random (MAR)”. As predictors we used the socio-demographic and outcome variables mentioned above at t_0_, t_1_, t_2_, t_3_, and t_4_. For all analyses reported below, we used the statistical software Mplus 6.11 (www.statmodel.com) [18].

### Statistical analysis

First, we considered characteristics of the intent-to-treat sample (N = 155) and described them with means, SDs, and frequencies.

Selection effects were analyzed using Chi-square-test, regarding age, gender, and pre-measures of pain-related and psychological variables.

To test the first hypothesis, we compared post-treatment means of immediately-treated patients (N = 89) with post-waiting time means of waiting group patients (N = 66). The outcome means were adjusted for gender, age, pain intensity, depression, anxiety, and well-being. These confounders were assessed at pre-treatment and pre-waiting time, respectively. The means were estimated using a generalized analysis of covariance, incorporating the treatment variable, confounders, and the interactions of treatment variables and each confounder.

For the second hypothesis we estimated the average of pain intensity, depression, anxiety, and well-being for each assessment. The means refer to the intent-to-treat sample (N = 155). Missing data were imputed (see above). To standardize the changes of pain intensity and the psychological variables, we divided the mean difference (e.g. well-being mean of t_0_ and t_1_) by the standard deviation of the variable under study at t_1_.

Using a structural equation model and the intent-to-treat sample (N = 155), we studied the associations between healthcare utilization before the MMT and post-treatment measures (third hypothesis). Dependent variables were post-treatment measures of pain intensity, general well-being, anxiety, and depression. The explanatory variables were healthcare utilization (number of consulted medical specialist groups, number of types of treatment received and long-term opioid treatment). Furthermore, we controlled for pain intensity, general well-being, anxiety, and depression assessed at the beginning of the therapy.

## Results

### Subjects: Descriptive statistics

The average age of the intent-to-treat sample (N = 155) was 58.3 (SD = 10.4) years and 55.6% of the patients were female. Before attending the pain center, patients, on average, had consulted 4 (SD = 2.0) different medical specialist groups and had received 6 (SD = 2.4) different types of treatment. Patients attended the pain center with a pain intensity on the NRS (0–10) = 6.5 (SD = 1.9). The average pain duration was 18.1 (SD = 14.4) years. Of the patients, 42.4% reported opioid medication lasting longer than three months, at least. The depression scale (ADS) revealed scores slightly above the normal range with M = 23.7 (SD = 9.5). General well-being (MFHW) showed decreased levels (M = 9.6, SD = 8.1). Anxiety was within the normal range HADS = 9.8 (SD = 4.4). Table 1 also provides information about socio-demographic, pain-related, psychological, and healthcare utilization variables separated for waiting group patients (N = 66) and immediately-treated patients (N = 89).

**Table 1:** Socio-demographic, pain-related, psychological and healthcare utilization measures for MMT group sample (waiting group patients and immediately-treated patients.

	Pre-Treatment Variable	Waiting Group Patients (N = 66)		Immediately Treated Patients (N = 89)		MMT Group (N = 155)	
		mean	SD	mean	SD	mean	SD_pooled_
**SOCIO-DEMO-GRAPHIC**	**Gender (female)**	**57.6%**		**53.9%**		**55.6%**	
	**Age (years)**	**58.0**	**9.7**	**58.6**	**10.8**	**58.3**	**10.4**
**PAIN**	**Pain Duration (years)**	**17.9**	**14.9**	**18.3**	**13.9**	**18.1**	**14.4**
	**Pain Intensity (NRS)**	**6.6**	**1.6**	**6.4**	**1.8**	**6.5**	**1.9**
**PSYCHO-LOGICAL**	**General Well-Being (MFHW)**	**10.7**	**7.9**	**8.8**	**8.2**	**9.6**	**8.1**
	**Anxiety (HADS)**	**9.8**	**4.1**	**10.00**	**4.5**	**9.8**	**4.4**
	**Depression (ADS)**	**21.6**	**8.8**	**23.5**	**10.0**	**23.7**	**9.5**
**HEALTH CARE USE**	**Opioid medication (>3 month)**	**40.0%**		**45.5%**		**42.4%**	
	**Consulted medical specialist groups (range 0–11)**	**3.9**	**2.1**	**4.0**	**1.9**	**3.9**	**2.0**
	**Different types of treatment used (range 0–13)**	**5.8**	**2.5**	**6.1**	**2.3**	**5.9**	**2.4**

Selection effects were analyzed and revealed no significant differences between waiting group patients and immediately-treated patients with respect to age, gender, pre-test pain intensity, depression, anxiety, and general well-being (χ^2^ = 4.478, df = 6, p = .577).

### Comparison of the waiting group patients and immediately-treated patients

Table 2 shows the adjusted post-waiting time means of the waiting group patients (N = 66) and post-treatment means of immediately-treated patients (N = 89). For all scales, significant group differences were found. The average treatment effect for general well-being was large (ES = 0.92). The effects for depression and pain intensity were moderate (ES = -0.79 and ES = -0.76, respectively). The effect for anxiety was small (ES = -0.46). The direction of all effects was in the expected direction. The symptoms at end of MMT and the waiting period, respectively, were lower in the intervention group than in the control group.

**Table 2:** Adjusted means for waiting group patients (N = 66) and immediately-treated patients (N = 89), and comparison statistics.

Outcome Variable	Waiting group Patients (N = 66)		Immediately Treated Patients (N = 89)		Group Difference			
	mean	(SD)	mean	(SD)	diff	p	SD_pooled_	ES
**Pain Intensity**	**6.5**	**(1.6)**	**5.0**	**(1.9)**	**-1.45**	**0.000**	**2.0**	**-0.76**
**General Well-Being**	**12.5**	**(7.3)**	**20.0**	**(8.5)**	**7.50**	**0.000**	**8.2**	**0.92**
**Anxiety**	**9.1**	**(2.9)**	**7.3**	**(3.8)**	**-1.78**	**0.003**	**4.1**	**-0.46**
**Depression**	**22.0**	**(8.1)**	**14.9**	**(8.5)**	**-7.10**	**0.000**	**9.0**	**-0.79**

S: standard; SD_pooled_: pooled standard deviationdiff: difference of group means; p: p-value for the hypothesis H_0_: diff = 0;ES: effect size (ES = diff / SD_pooled_)

### Longitudinal changes in pain intensity and psychological variables

To test the second hypothesis, we analyzed changes in the outcome variables up to the twelve-month follow-up (see Table 3). Patients significantly improved in pain intensity and psychological variables after MMT (pre-post). The effect sizes ranged from large effects (general well-being, ES = 1.06) to moderate effects (pain intensity, ES = -0.74; depression, ES = -0.77; and anxiety, ES = -0.55). The effects of MMT on pain intensity and psychological variables were reduced at the three-month follow-up. Nevertheless, they were still significant with respect to pre-measures (pain intensity, ES = -0.48; general well-being, ES = 0.78; anxiety, ES = -0.34; and depression, ES = -0.30). This tendency continued until the twelve-month follow-up. The effect sizes were small for anxiety and pain intensity (ES = -0.22 and ES = -0.20, respectively). For general well-being the effect size was moderate with ES = 0.61. Depression scores at the twelve-month follow-up reached the level of pre-treatment (ES = -0.11).

**Table 3:** Time course of pain intensity (NRS), general well-being (MFHW), anxiety (HADS) and depression (ADS).

	Begin of Waiting Time (t_0_)		Pre MMT (t_1_)		Post MMT (t_2_)		Follow-up at 3months (t_3_)		Follow-up at 12 months (t_4_)	
	**mean**	**(SD)**	**mean**	**(SD)**	**mean**	**(SD)**	**mean**	**(SD)**	**mean**	**(SD)**
**Pain Intensity**	**6.6**	**(2.5)**	**6.5**	**(2.5)**	**5.1**	**(2.5)**	**5.6**	**(2.5)**	**6.3**	**(2.5)**
**General Well-Being**	**10.8**	**(12.4)**	**10.7**	**(8.7)**	**19.7**	**(8.7)**	**17.3**	**(13.7)**	**15.9**	**(14.9)**
**Anxiety**	**9.8**	**(6.2)**	**9.4**	**(3.7)**	**7.1**	**(3.7)**	**8.0**	**(6.2)**	**8.5**	**(6.2)**
**Depression**	**21.7**	**(13.7)**	**22.5**	**(10.0)**	**15.1**	**(8.7)**	**19.3**	**(14.9)**	**20.7**	**(17.4)**

(N = 155 persons at each time point)

During the waiting period (t_0_ to t_1_), no significant changes occurred in primary and secondary outcome variables.

### Impact of pre-admission healthcare utilization on MMT outcome

**Fig 2:**
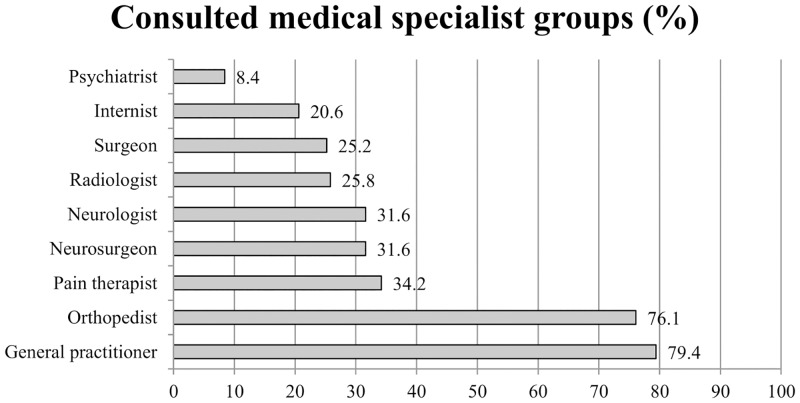
Consulted medical specialist groups in percent (%).

Patients first consulted general practitioners (79.4%) and orthopedists (76.1%). Pain therapists (34.2%) and neurologists/neurosurgeons (31.6%) were consulted in third and fourth place (Fig 2).

**Fig 3:**
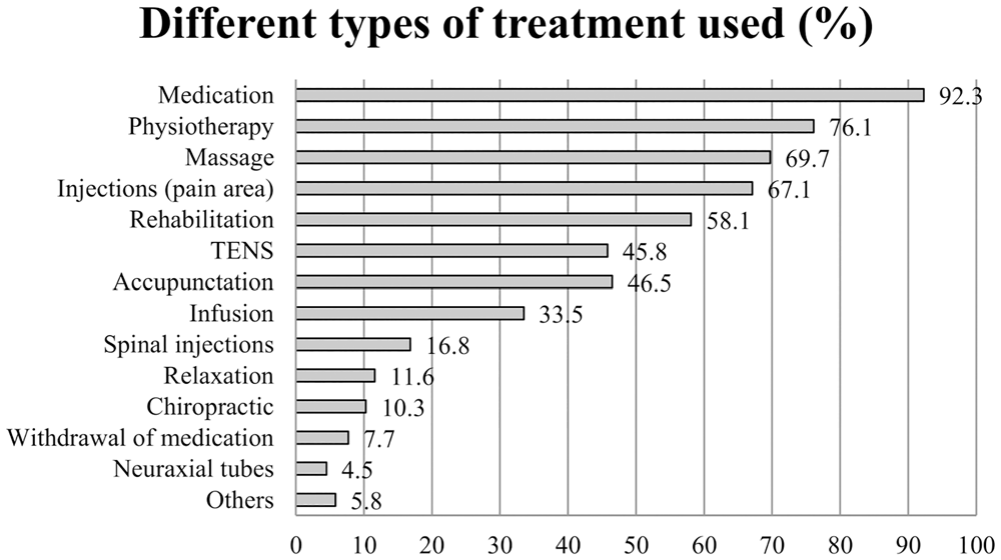
Different types of treatments used in percent (%).

With respect to the kind of treatment (Fig 3), nearly all patients received some kind of pain medication (92.3%). In the second line physiotherapy (76.1%) and massage (69.6%) were received.

Correlation analyses revealed no significant association between healthcare utilization variables and pre-treatment pain intensity, general well-being, depression and anxiety (Pearson’s r ranged from r = 0.001 and r = 0.08).

**Fig 4:**
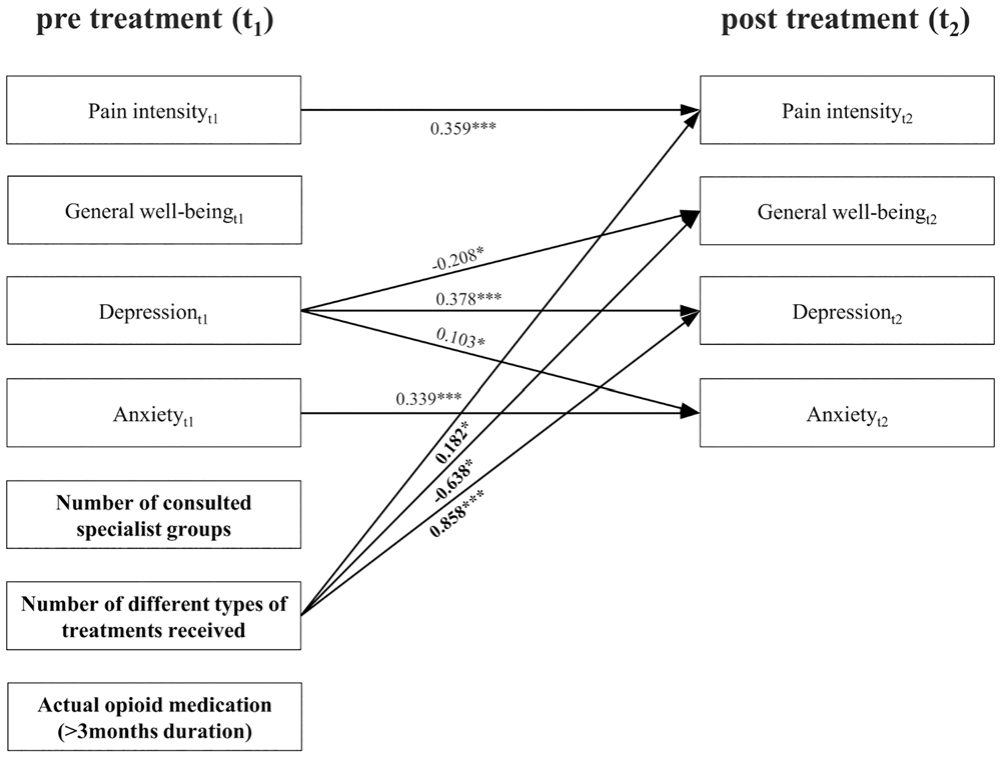
Path diagram of associations between pre-admission healthcare utilization, pre-treatment pain intensity, general well-being, depression, anxiety and post-treatment measures. Unstandardized regression coefficients, * p < 0.05, ** p < 0.01, *** p < 0.001; non-significant associations and covariance are not shown.

Using a structural equation model, we analyzed the associations between healthcare utilization before the MMT and post-treatment measures (pain intensity, general well-being, depression, and anxiety). The corresponding path diagram is shown in Fig 4. It can be seen that with increasing numbers of different types of treatments received before MMT, lower values of general well-being after therapy can be expected (unstandardized regression coefficient λ = -.184, p = .030). Furthermore, depression ratings (λ = .244, p = .000) and pain intensity after therapy (λ = .201, p = .020) increased with the number of treatment types undertaken before therapy. Post-treatment anxiety could not be predicted based upon healthcare utilization. The number of medical specialist groups consulted, as well as long-term medication with opioids, had no significant influence on MMT outcome (Fig 4).

## Discussion

In our study we found evidence for the effectiveness of a multimodal therapy for patients with chronic low back pain. Primary and secondary outcomes at end of MMT and the waiting period, respectively, were lower in the intervention group than in the control group. The effect sizes ranged from high (general well-being) to moderate (pain intensity and depression) and small (anxiety). These results are consistent with other evaluation studies of MMT with a treatment intensity of a minimum of 100 hours and a functional restoration approach (see e.g. [5]). According to reviews which reported mostly small to moderate effects of MMT on pain intensity and disability (e.g., [19, 20]) we found a large effect on well-being and moderate effects on pain intensity and depression. At pre-treatment, a multidisciplinary team assessed the suitability of MMT in our study. A specific motivation of the individual patient to undergo an activating multimodal therapy was explicitly considered within this assessment. Careful pre-treatment interdisciplinary assessment has been shown to improve both MMT and other pain treatment outcomes [21, 22].

Furthermore, we found long-term effects of the MMT, although the post-treatment effect decreased at the three-month follow-up in relation to pain intensity and psychological variables. However, pain intensity, depression, anxiety and well-being at the three-month follow-up were significantly better than at the beginning of the MMT. The effect sizes were at least small (pain intensity, anxiety, and depression) and/or moderate (general well-being). This tendency continued up to the twelve-month follow-up. The effect sizes remained small for pain intensity and anxiety, and moderate for general well-being. In contrast, depression scores at the twelve-month follow-up had increased nearly up to the pre-treatment level. General well-being turned out to be the most salient factor through to follow-up after twelve months. It seems that patients could improve their handling of the painful condition due to MMT and could better accept high levels of pain intensity.

Patients consulting the pain center showed a high amount of pain-related burden. They also reported a large number of consulted medical specialist groups as well as types of pre-admission treatments. First, patients usually consulted a general practitioner (79.4%) or an orthopedist (76.1%). In the third and fourth place neurologists/neurosurgeons (31.6%) and pain therapists (34.2%) were consulted. This result is in line with other German and European studies (e.g., [23–25]). In Europe, chronic pain patients consult more general practitioners (70%) than orthopedists (27%) [24]. This might be related to a “gate-keeping system” in some European countries. In contrast, the German system provides barrier-free access to any medical specialist. This might explain the high rate of orthopedists consulted [23, 25].

With regard to pre-admission healthcare utilization we found that the number of patients who had consultations with pain management specialists was 34.2% in the sample. Breivik *et al*. (2006) reported much lower rates for Germany. Among the European countries, Germany and Norway showed the fewest number of pain management specialist consultations, with 10% and 8% respectively. Italy and France were in the top positions with 42% and 40%, respectively, which represents chronic pain patients with at least one consultation [24]. The elevated rates in our study might be due to the special population of patients with long-term disabling low back pain.

The treatment that patients received before attending the pain center predominantly consisted of medication, physiotherapy, massage, and local injections. These are almost exclusively passive procedures, which overall are not recommended by the current guidelines for the management of low back pain (e.g., the German national guidelines, [7]). Massage, for example, is explicitly not recommended for chronic pain conditions [7, 26], but still seems to be a common treatment in Germany. Of German chronic pain patients, 46% reported having received massage treatment, in contrast to 15% in the UK [24]. Physiotherapy reflects a wide range of different procedures, ranging from passive mobilization to supervised active exercises. Accordingly, it is impossible to assess the conformity of physiotherapeutic treatment with the existing guideline recommendations. An earlier study with participants of the New German Back School [27] revealed a similar pattern of predominantly passive treatments in the history of the patients. In this study, participants with relatively low pain-related burden were surveyed in a secondary preventive setting. The most common procedures participants reported were also massage (39.8%), medication (30.7%), and local injections (25%). Physiotherapy was in the fourth position with 23.9%. Taken together, the results indicate a general pattern of high utilization of passive treatments in the management of low back pain. This is in opposition to current guideline recommendations (e.g. [7]).

At last associations between the effectiveness of MMT and pre-admission healthcare utilization were examined. Results of the structural equation model indicate that, with an increasing number of different types of treatments received before MMT, a more negative treatment outcome has to be expected in pain intensity, general well-being, and depression after therapy. This corresponds with findings of previous researchers [10, 12]. This result leads to the conclusion that MMT patients should be treated with MMT in an earlier state of the pain treatment process. In Germany, actually, the opposite way is required by the current guidelines. All outpatient treatment opportunities for low back pain have to be exploited before attending MMT.

It could be considered whether patients with an elevated use of different treatment types might have higher scores in pain-related and psychological factors already prior to treatment. For our analyses this assumption does not hold because we statistically-controlled for baseline pain-related and psychological factors. Furthermore the generalizability is limited by the fact that we considered baseline measures only. It is still possible that these patients will have worse pain conditions in earlier stages of their treatment history. On the other hand, patients with elevated numbers of treatment types might generally have less benefit from pain treatments, including MMT. This leads to the suggestion that patients would benefit more from attending MMT in an earlier stage of healthcare utilization.

Several limitations must be noted. The generalizability of the results is potentially limited. The sample consisted of consecutively-included patients from only one pain clinic.

Due to the waiting setting (treatment as usual), there was no real control group for the whole study period. Therefore no comparison with other treatments or pain conditions was possible. The waiting subgroup was a naturally formed group, and no randomization could be applied. But we found no differences with regard to pre-test values between waiting group and intervention group. Additionally, selection effects were handled by controlling pre-treatment measures in the statistical model and computation of adjusted means. A further limitation refers to the assessment of pain disability which is an important outcome measure in therapy with pain conditions (e.g., [28]). Unfortunately, pain disability could not be considered for evaluation caused by a questionnaire fault in the Pain Disability Index scales at follow up. However, we were able to assess pain intensity as a pain-related outcome measure.

### Conclusion

The treatment effects of the MMT suggest that patients with chronic low back pain benefit from the intensive three-week inpatient MMT. This underlines the importance of treating low back pain with relation to physical as well as psychological aspects. The association between treatment effect and number of different pre-admission pain treatment types suggests that patients would benefit more from attending MMT in an earlier stage of healthcare utilization, thus avoiding elevated use of multiple treatment types.

## Supporting Information

S1 Data SPSS Data file. (SAV)
